# Gun-shot Wound of the Heart

**Published:** 1867-12

**Authors:** V. S. Lindsley

**Affiliations:** the University of Nashville.


### ARTICLE IX.

### *Gun-shot Wound of the Heart.*

By V. S. Lindsley, M.D., of the University of Nashville.

The following is a curious case. The evidences pointing to a wound of the heart are so strong, amounting almost to a certainty, that to call it a gunshot wound of the heart hardly seems a venture. There are cases on record whose history did not show such clear and pointed evidences of a wound of this organ during life, and yet an examination after death revealed that the heart had not only been wounded, but its cavity penetrated.

L. P., æt. 24 years, in full health and in the enjoyment of a vigorous constitution, was loading and adjusting a small four-barrelled Sharp's pistol on Saturday, the 22nd of May, 1865. The barrels of the pistol joined the stock by a hinge joint, and fastened by a spring. Holding the barrels in his left hand, he inserted the cartridges, and, thinking everything right, sprung the barrels to their place. As he did so one of the cartridges exploded and the ball entered his chest between the fourth and fifth cartilages, one-half inch to the left of the sternum, and on a line with the left nipple, the direction of the ball being oblique from right to left. The ball was of trifling size, being about 1-5 of an inch in diameter, and perhaps 2-5 in length. His first idea was that the wound would be fatal, and desiring to seek aid, he repaired immediately to a physician's office near by and fainted just after reaching the room.

From the time of the explosion of the cartridge to the fainting, patient describes the pain as "excruciating and very acute in the region of the heart, as if a sharp knife had been driven in and worked back and forth." A feeling of dizziness was soon added to the pain.

It was at this juncture I first saw him, and to all appearances it seemed likely that death would soon close the scene. The shock had been powerful, the syncope was very extreme, pulsations at the wrist were absent, and respiration was almost entirely suspended. The hemorrhage from the wound was very slight.

Drs. Briggs and Jennings had been called in and were now present. The case was thought hopeless. There were signs strongly indicating wound of the heart, viz.: the direction of the wound, the extreme anxiety, dyspnœa, syncope, and probably internal hemorrhage. Soon, however, as if awakened by some powerful influence, the patient was thrown into the most violent muscular exertion, so that it required four men to hold him in bed. Strange to say he knew *no* one, and uttered only inarticulate sounds. Chloroform was immediately ordered and administered with the happiest effect. The patient lay at rest, rescued from his tormentor, and breathed quietly but very slowly. As yet there was no pulsations at the wrist and the heart beat feebly. He was now removed to his room, and full doses of morphia administered through the day. The surface of the body remained cool and it was not until eight o'clock at night, that he regained his natural warmth and consciousness.

Sunday.—The syncope following the wound, together with the morphia seemed to have obliterated entirely all recollection of the events of Saturday. When he complained of pain in the chest, he was told it was the result of a fall. Lying in the horizontal position was so painful, that he had to be propped in a half reclining posture. There was no coughing or spitting of blood, nor were his lungs in the least affected.

To control the pain ready to burst forth whenever the anodyne influence subsided, heavy doses of morphia were regularly given.

On Tuesday calomel was added to hold in check any arising inflammation.

Thursday, marked symptoms of pericarditis ensued. Up to this time there was not the slightest intermission of the very intense pain which commenced just after the infliction of the wound ; the patient himself insisting upon the repetition of the anodyne. Thursday evening a blister was applied over the region of the heart. Its effect almost instantaneous ; in the language of the patient, " it seemed that some powerful agency was at work drawing the pain from my heart. After that, the the acute pain, though great at times, was often absent. The pain was always at the heart."

For the next seven or eight days his condition gave lively hope of recovery, but on the fourteenth night after the wounding, there was a return of the pain with all its former violence. The blister was reapplied over the region of the heart, and again gave marked relief. Mercury with opium was given every few hours and continued for a number of days for the purpose of inducing ptyalism. But it utterly failed to produce this effect. During the whole time, and he took a large quantity of mercury, there was not the least evidence that the remedy had produced its peculiar effect upon the constitution.

The severity of the pain gradually relented, stimulants and supportive treatment were administered, and the patient had another respite of about seven days. On the twenty-first night the accession of pain was so great, that he thought death was near, and so did his physician. The pain was of the most violent character, originating at the heart and radiating through all parts of the chest. His distress was extreme, and so powerfully did it depress his vital powers, that the heart almost refused to act, the wrists were pulseless, and stimulants were required in liberal amount. The feet were bathed and anodynes again given.

After this night he became more comfortable, took stimulants more kindly, and gradually improved until he became convalescent. The fifth week from date of wound, he rode out in a carriage, and in seven months he was sound and well, able to attend to all his duties as usual.

There is an interesting psychological fact connected with this case. The patient never recalled any of the circumstances of the accidental "going off" of the pistol, nor even the fact of having wounded himself at all, until it was told him after his recovery. When it was mentioned, the whole affair flashed across his mind in an instant, he remembered every thing. During his entire illness the impression had been kept up, that the pain from which he suffered so terribly, was the result of a fall. No doubt the morphia of which he took so largely, tended to prevent his mind from reverting to the accident.

The probability is, that the ball passed through the intercostal muscles, the pericardium, and entered the muscular wall of the heart, but did not pass its cavity. It is now three years since the accident, and the gentleman enjoys as good health, with perfect action of the heart, and freedom from pain, as if nothing had ever occured.—*Nashville Journal of Med. and Surg.*

---

